# Optimising the number of cores for magnetic resonance imaging‐guided targeted and systematic transperineal prostate biopsy

**DOI:** 10.1111/bju.14865

**Published:** 2019-08-01

**Authors:** Nienke L. Hansen, Tristan Barrett, Thomas Lloyd, Anne Warren, Christina Samel, Ola Bratt, Christof Kastner

**Affiliations:** ^1^ CamPARI Prostate Cancer Group Addenbrooke's Hospital University of Cambridge Cambridge UK; ^2^ Department of Diagnostic and Interventional Radiology Faculty of Medicine University Hospital of Cologne Medicine and University Hospital of Cologne Cologne Germany; ^3^ Department of Radiology Cambridge University Hospitals Cambridge UK; ^4^ Department of Urology Cambridge University Hospitals Cambridge , UK; ^5^ Department of Pathology Cambridge University Hospitals Cambridge , UK; ^6^ Institute of Medical Statistics and Computational Biology (IMSB) University Hospital of Cologne Cologne Germany; ^7^ Department of Urology Sahlgrenska Academy Gothenburg University Gothenburg Sweden

**Keywords:** transperineal, magnetic resonance imaging, MRI‐TRUS fusion, prostate biopsy, #ProstateCancer, #PCSM

## Abstract

**Objectives:**

To assess cancer detection rates of different target‐dependent transperineal magnetic resonance (MR)/ultrasonography (US) fusion‐guided biopsy templates with reduced number of systematic cores.

**Patients and Methods:**

Single‐centre outcome of transperineal MR/US fusion‐guided biopsies of 487 men with a single target MR imaging (MRI) lesion, prospectively collected between 2012 and 2016. All men underwent transperineal targeted biopsy (TB) with two cores, followed by 18–24 systematic sector biopsies (SB) using the Ginsburg protocol. Gleason score ≥7 prostate cancer detection rates for two‐core TB, four‐core extended TB (eTB), 10‐ to 20‐core saturation TB (sTB) including cores from sectors adjacent to the target, and 14 core ipsilateral TB (iTB) were compared to combined TB+SB.

**Results:**

Cancer was detected in 345 men and Gleason score 7–10 cancer in 211 men. TB alone detected 67%, eTB 76%, sTB 91% and iTB 91% of these Gleason score 7–10 cancers. In the subgroup of 33 men (7% of cohort) with an anterior >0.5 mL highly suspicious MRI lesion and a prostate volume ≤45 mL, four‐core eTB detected 31 of 32 cancers (97%) and all 26 Gleason score 7–10 cancers.

**Conclusion:**

sTB detected Gleason score 7–10 cancer in 25% more of the men than a two‐core TB approach, and in almost as many men (91%) as the 20–26‐core combined TB+SB, while needing only 10–20 cores. A four‐core extended TB may suffice for large, highly suspicious anterior lesions in small or slightly enlarged prostates.

**Abbreviations**
ISUP: International Society of Urological Pathology
mpMRI: multiparametric MRI
PI‐RADS: Prostate Imaging Reporting and Data System
PROMIS: PROstate MRI Imaging Study
SB: sector biopsy
(e)(i)(s)TB: (extended) (ipsilateral) (saturation) targeted biopsy
US: ultrasonography

## Introduction

Multiparametric MRI (mpMRI) of the prostate is increasingly used for high‐risk patients with negative systematic biopsy or biopsy‐naïve patients, to target biopsies either by cognitive or fusion approaches 1, 2. This has led to debate whether targeted biopsies (TB) alone are sufficient or additional systematic biopsies (TB+SB) remain necessary 1, 2, 3, 4, 5, 6. The high negative predictive value of prostate mpMRI 7 suggests extensive SBs may not always be required in addition to TB cores in men with suspicious MRI lesion/s. Transrectal MR/ultrasonography (US) image fusion with 5–6 target cores has been shown to detect up to 90% of cancers found at prostatectomy 8 and 1–4 target cores to detect, correctly locate, and identify the primary Gleason pattern in >90% of index tumours 9. The recent PRECISION trial suggests that four biopsy cores targeted to suspicious MRI lesions, outperform a standard 10–12‐core transrectal SB 4. Although targeted biopsy alone has advantages, this approach may lead to an unacceptable proportion of missed high‐grade cancers 5, 10, 11, 12. Conversely, systematic cores increase detection of clinically insignificant cancer 3, 4, 6, and identifying significant cancers with minimal over‐diagnosis of insignificant disease is the clinical goal. A compromise may be to reduce the number of systematic cores. Recently, Bryk et al. 13 reported that adding six ipsilateral systematic biopsies to transrectal TB substantially increased the detection of clinically significant cancer, whereas contralateral systematic biopsies mainly detected insignificant cancer. Calio et al. 14 reported that four TB cores better predicted Gleason score at prostatectomy than a single TB core. Similarly, the addition of four perilesional cores (‘focal saturation’) improved the detection of clinically significant cancer on MRI‐guided in‐bore biopsy 6. In addition, we know that MRI tends to substantially underestimate histopathological volumes 15. These results suggest that systematic cores may be avoided if the target lesion and adjacent prostate are sufficiently sampled. This approach could reduce over‐diagnosis of insignificant cancers, but also reduce morbidity, pathologist workload and, potentially, the need for general anaesthesia for transperineal targeted biopsies.

The transperineal Ginsburg MR/US‐fusion biopsy protocol currently includes two TB and 18‐24 SB cores 16. This protocol has been validated to detect 97% of significant cancers in men undergoing prostatectomy 17. The two TB cores alone detected 80% of the cancers, in line with other reports suggesting that two TB cores alone were inadequate 18, 19, 20, 21. The aim of the present study was to model the accuracy of different target‐dependent transperineal MRI/US fusion‐guided biopsy templates with reduced number of systematic cores.

## Patients and Methods

### Study Population ‐ Inclusion Criteria and Data Collection

In all, 690 men underwent transperineal MR/US fusion‐guided biopsies following positive mpMRI at Cambridge University Hospitals Trust between March 2012 and June 2016. To avoid overlap of lesions, the present study only included the 490 men with a single MRI lesion identified. Three were excluded because of previous brachytherapy. Of the remaining 487, 122 (25%) had no previous biopsy, 214 (44%) had a previous negative biopsy, and 152 (31%) were patients on active surveillance for low‐grade cancer. Men on active surveillance were included because the Gleason score ≥7 cancer detection is similar in men without previous cancer and in men on active surveillance for Gleason score 6 cancer 22. Data were collected prospectively and reported according to the Standards of Reporting for MRI‐targeted Biopsy Studies (START) to describe the study population, MRI sequences, image registration and MRI reporting 23.

### Ethics Approval

All men were counselled about the risks of the procedure and provided informed consent, including permission to use their clinical data for research. The study was approved as a service evaluation by the local audit and ethics committees at Cambridge University Hospitals Trust.

### MRI

Men underwent prostate MRI on a 1.5‐T MR450 or 3.0‐T Discovery MR750 HDx (GE Healthcare, Waukesha, WI, USA) with an 8–32 channel surface phased‐array coil. Axial fast spin‐echo T1‐weighted images of the pelvis, along with T2‐weighted fast recovery fast spin‐echo images of the prostate were acquired in the axial (slice thickness 3 mm; gap 0–1 mm), sagittal, and coronal planes. Axial diffusion‐weighted imaging was performed using a spin‐echo echo‐planar imaging pulse sequence with slice thickness 3–4 mm; gap 0 mm (*b*‐values: *b*‐150, *b*‐750, *b*‐1400, *b*‐2000 s/mm^2^); apparent diffusion coefficient maps were automatically calculated.

### Image Analysis

MR images were prospectively reported by one of two uroradiologists with >5 years’ experience of reading prostate MRI using a Likert scale, based on the Prostate Imaging Reporting and Data System (PI‐RADS) structured scoring criteria developed by the European Society of Urogenital Radiology (ESUR). The contours of Likert 3–5 lesions were drawn on the Biopsee™ MRI‐TRUS fusion biopsy platform (Medcom, Darmstadt, Germany), which also measured three‐dimensional lesion volume.

### Biopsy

The Biopsee MRI/TRUS‐fusion biopsy system version 1 or 2 (Medcom) was used for all biopsies. All men had 24 systematic (SB) cores taken according to the Ginsburg protocol, using a spring‐loaded biopsy gun with an 18‐G needle 16, 24. Two biopsy cores were taken from each lesion prior to SB cores, which include two biopsy cores sampled from each of 12 sectors, starting with the anterior sectors. All procedures were done by one of three urologists with several years’ experience of transperineal biopsy using the Biopsee MRI/TRUS‐fusion biopsy system.

### Histopathology

The Gleason score of any tumour present was assessed according to the International Society of Urological Pathology (ISUP) 2005 recommendations and assigned per site, in addition to the number of positive cores and the percentage involvement of the tissue 25. According to the 2016 prostate cancer grading system, Gleason Score 6 cancers are regarded as clinically insignificant, equivalent to Grade Group 1, and clinically significant cancers were defined as Gleason Score 7–10, equivalent to combined Grade Groups 2–5 26. All biopsy specimens were reported by a uropathologist and reviewed by a second uropathologist prior to discussion at multidisciplinary team meetings. The final histology result provided data for this study.

### Definition of Biopsy Templates

**Figure 1:**
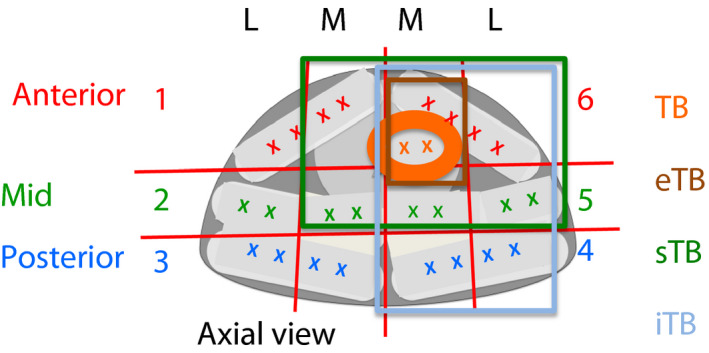
Ginsburg scheme and possible modifications. Current Ginsburg scheme with two target cores and 24 systematic cores; four‐core eTB; sTB with two target cores, two target‐zone cores, 6–16 systematic cores in adjacent sectors depending on localisation of target; iTB with two target cores, two target‐zone cores, and 10 ipsilateral systematic cores.

The different biopsy templates were defined as follows:

TB = 'targeted biopsy' = two cores
eTB = ‘extended TB’ = TB plus two systematic cores in target sector (four cores total)
sTB = ‘saturation TB’ = two cores from the target plus two cores from the target sector plus two cores from each of the adjacent sectors, as defined by the Ginsburg protocol (six cores for corner sectors 1L, 3L, 4L, 6L; 10 cores for peripheral sectors 1M, 2L, 3M, 4M, 5L, 6M; 16 cores for central sectors 2M, 5M, that is 10–20 cores total depending on location)
iTB = ‘ipsilateral TB’ = TB plus ipsilateral biopsy of 12 systematic sector cores (14 cores total)
TB+SB = standard TB plus 18–24 systematic sector cores (20–26 cores total) (Fig. 1).

### Statistical Analysis

The data were analysed using the IBM Statistical Package for the Social Sciences (SPSS®), version 23 (SPSS Inc., IBM Corp., Armonk, NY, USA). The detection of any cancer and Gleason score 7–10 (Grade Group 2–5) cancer on TB, eTB, sTB, and iTB was compared with the detection on TB+SB as the ‘gold standard’. The 95% CIs of detection rates were calculated and intergroup differences between sensitivities were considered statistically significant if the 95% CIs did not overlap. Subgroup analyses were performed for sector location, Likert probability of MRI, prostate volume, and lesion‐volume groups. Prostate volume was dichotomised at a threshold of 45 mL and MRI lesion volume of 0.5 mL using the median prostate and lesion volume of the study population and clinical utility as guidance. Differences between subgroup proportions were compared with the Fisher's exact test using the Freeman‐Halton extension. For comparison of sTB and iTB, McNemar tests were performed. A *P* < 0.05 was considered statistically significant. All analyses were of an explorative nature, therefore no adjustment for multiple testing was performed.

## Results

The clinical characteristics of the 487 included men are shown in Table 1 and the distribution of Gleason scores on biopsy in Table S1.

**Table 1:** Clinical characteristics of the patients included in the study

Characteristic	Value
Number of patients	487
Proportion first/previous benign TRUS/active surveillance, *n*	121/214/152
Age, years, median (IQR)	66 (60–69)
Median (IQR)	
Pre‐biopsy PSA level, ng/mL	7.2 (5.0–10.5)
P Prostate volume, mL	46 (34–73)
PSA density, ng/mL/mL	0.14 (0.09–0.23)
Lesion volume, mL	0.50 (0.28–1.00)
TBs per patient, *n*	2 (2–2)
Total biopsies per patient, *n*	26 (26–27)

IQR, interquartile range.

### Comparison of Different Biopsy Models

**Figure 2:**
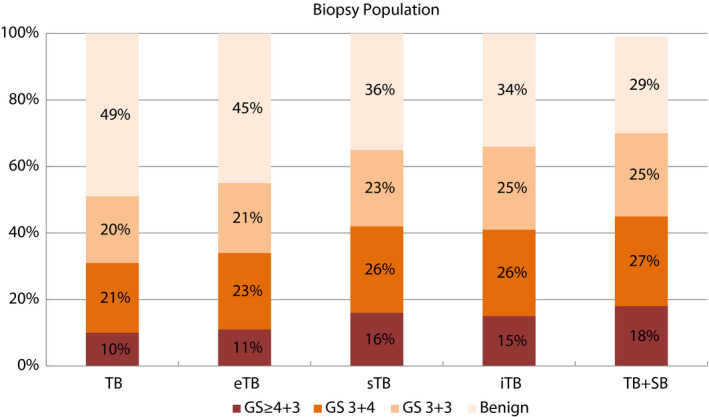
Cancer detection rates by different biopsy models (% of 487 men in study population). Targeted fusion biopsies with two cores showed generally low sensitivity with 67% of Gleason score (GS) 7–10 prostate cancer, eTB with four cores detected 76%, and both the 14‐core iTB and the 10–20‐core sTB detected 91% of clinically significant prostate cancer detected by combined 20–26‐core TB+SB reference, with slightly higher detection of Gleason score 3+3 prostate cancer with the iTB.

The gold standard combination of TB+SB detected cancer in 345 (71%) and Gleason score ≥7 cancer in 221 (45%) men. The cancer detection by the different templates with reduced core numbers is shown in Fig. 2 and Table 2. As expected, detection increased with increasing systematic core number: two‐core TB detected only 67% of the Gleason score ≥7 cancers, eTB only 76%, whilst the sTB and iTB templates both detected >90% of Gleason score ≥7 cancers. Comparing sTB and iTB, there was no statistically significant difference between both biopsy schemes in detection of any or Gleason score 7–10 cancer (*P* = 0.093 and *P* = 1.0, respectively). The reduction of Gleason score 6 cancer detection was less marked: only 5% fewer men were diagnosed with Gleason 3+3 cancer with two‐core TB. Regarding feasibility of an eTB model for certain subgroups of men, the four‐core eTB was equivalent to iTB and sTB detecting all 26 Gleason ≥7 cancers (95% CI 89–100%) in 33 men with a >0.5 mL Likert 5 lesion in the anterior sectors of a ≤45 mL prostate (Table S3).

**Table 2:** Cancer detection rates of different biopsy models

	TB			eTB			sTB			iTB			TB+SB	
	*n*	% of TB+SB	95% CI	*n*	% of TB+SB	95% CI	*n*	% of TB+SB	95% CI	*n*	% of TB+SB	95% CI	*n*	% of TB+SB
All														
Any prostate cancer	246	71	66.2–76.0	269	78	73.2–82.2	314	91	87.5–93.8	323	94	90.5–96.0	345	100
Gleason score ≥7	149	67	60.8–73.6	169	76	70.3–81.9	202	91	86.9–94.7	201	91	86.4–94.4	221	100

TB, two cores; eTB, four cores total; iTB, 14 cores total; sTB, 10–20 cores total depending on location; ; TB+SB, standard TB plus 18–24 SB sector cores (20–26 cores total).

### Comparison of Target Sector Location

eTB of lesions in the inner sectors of the prostate had a slightly lower sensitivity (59%, *P* = 0.131 when compared with 78% in the outer sectors; Tables S2 and 3). The eTB template had higher sensitivity in the anterior (83%) than in the mid and posterior sectors (71%) (*P* = 0.048).

**Table 3:** Detection rates by sector location

	TB			eTB			sTB			iTB			TB + SB	
	*n*	% of TB+SB	95%CI	*n*	% of TB+SB	95%CI	*n*	% of TB+SB	95%CI	*n*	% of TB+SB	95%CI	*n*	% of TB+SB
**Anterior sectors 1L 1M 6M 6L** **10–14 cores** ***N*** **= 200**														
Any prostate cancer	130	81	73.8**–**86.5	142	88	82.2–92.7	152	94	89.7–97.4	150	93	88.1–96.5	161	100
Gleason score ≥7	78	74	64.8–82.3	87	83	74.3–895	97	92	85.5–96.7	94	90	82.0–94.7	105	100
**Mid sectors** **2L 2M 5M 5L** **14**–**20 cores** ***N*** **= 105**														
Any prostate cancer	42	63	50.0–74.2.	44	66	53.1–76.8	62	93	83.4–97.5	62	93	83.4–97.5	67	100
Gleason score ≥7	27	60	44.3–74.3	29	64	48.8–78.1	42	93	81.7–98.6	41	91	78.8–97.5	45	100
**Posterior sectors 3L 3M 4M 4L** **10**–**14 cores** ***N*** **= 183**														
Any prostate cancer	74	63	53.8–72.0	83	71	61.8–79.0	100	86	77.8–913	111	95	89.2–98.1	117	100
Gleason score ≥7	44	62	49.7–73.2	53	75	62.9–84.2	63	89	79.0–95.0	66	93	84.3–97.7	71	100
***P*** **value**														
Any prostate cancer	**0.001**			**<0.001**			**0.032**			0.783				
Gleason score ≥7	0.108			**0.048**			0.666			0.787				

TB, two cores; eTB, four cores total; iTB, 14 cores total; sTB, ‘10–20 cores total depending on location; TB+SB, standard TB plus 18–24 systematic sector cores (20–26 cores total). Significant *P* values in bold.

### Comparison of Likert Probability of MRI

The sensitivity was generally low for the TB (46%) and eTB (57%) templates in men with Likert 3 lesions, indicating that these lesions often do not represent significant cancer, but the men are at increased risk of having cancer elsewhere in the prostate. The sensitivity was higher for Likert 4 (TB, 64%; eTB, 71%) and Likert 5 lesions (TB, 75%; eTB, 85%) (*P* = 0.011–0.002; Table 4).

**Table 4:** Detection rates by Likert score of MRI

	TB			eTB			sTB			iTB			TB+SB		
	*n*	% of TB+SB	95%CI	*n*	% of TB+SB	95%CI	*n*	% of TB+SB	95%CI	*n*	% of TB+SB	95%CI	*n*	% of TB+SB	PPV, %
**Likert 3** ***N*** **= 140**															
Any prostate cancer	33	49	36.8–61.8	38	57	44.0–68.8	52	78	65.8–86.9	54	81	69.1–89.2	67	100	48
Gleason score ≥7	13	46	27.5–66.1	16	57	37.2–75.5	25	89	71.8–97.7	23	82	63.1–93.9	28	100	20
**Likert 4** ***N*** **= 164**															
Any prostate cancer	82	71	61.5–78.8	89	77	68.0–84.1	105	91	83.7–95.2	111	96	90.2–98.6	116	100	71
Gleason score ≥7	47	64	51.5–74.4	52	71	58.5–80.3	63	85	75.0–92.3	66	89	79.8–95.2	74	100	45
**Likert 5** ***N*** **= 183**															
Any prostate cancer	131	81	74.0–86.6	142	88	81.6–92.3	157	97	92.9–99.0	158	98	93.8–99.3	162	100	89
Gleason score ≥7	89	75	66.0–82.3	101	85	77.2–90.8	114	96	90.5–98.6	112	94	88.3–97.6	119	100	65
***P*** **value**															
Any prostate cancer	**<0.001**			**<0.001**			**<0.001**			**<0.001**					
Gleason score ≥7	**0.011**			**0.002**			**0.025**			0.093					

TB, two cores; eTB, four cores total; iTB, 14 cores total; PPV, positive predictive value; sTB, 10–20 cores total depending on location; TB+SB, standard TB plus 18–24 SB sector cores (20–26 cores total). Significant *P* values in bold.

### Comparison of Prostate Volume and Lesion Volume

In smaller prostates (<45 mL) eTB detected 82% of the Gleason score 7–10 cancers (*P* = 0.039). In an analysis of the men with data on lesion volume, TB and eTB were less likely to detect Gleason ≥7 cancer in men with lesions <0.5 mL (TB, 55%; eTB, 69%) than in men with larger lesions (TB, 76%; eTB, 82%) (*P* = 0.002–0.0047; Tables 5 and 6).

**Table 5:** Detection rates by prostate volume

	TB			eTB			sTB			iTB			TB + SB	
	*n*	% of TB+SB	95%CI	*n*	% of TB+SB	95%CI	*n*	% of TB+SB	95%CI	*n*	% of TB+SB	95%CI	*n*	% of TB+SB
≤**45 mL** ***N*** **= 233**														
Any prostate cancer	150	79	72.0–84.1	160	84	77.8–88.7	177	93	88.0–95.9	182	95	91.2–97.8	191	100
Gleason score ≥7	88	75	65.7–82.1	97	82	74.1–88.6	109	92	86.0–96.5	110	93	87.1–97.0	118	100
>**45 mL** ***N*** **= 254**														
Any prostate cancer	96	62	54.2–70.0	109	71	62.9–77.8	137	89	82.9–93.4	141	92	86.0–95.4	154	100
Gleason score ≥7	61	59	49.1–68.8	72	70	60.1–78.5	93	90	82.9–95.2	91	88	80.5–93.8	103	100
***P*** **value**														
Any prostate cancer	**0.001**			**0.004**			0.259			0.186				
Gleason score ≥7	**0.021**			**0.039**			0.636			0.244				

TB, two cores; eTB, four cores total; iTB, 14 cores total; sTB, 10–20 cores total depending on location; TB+SB, standard TB plus 18–24 SB sector cores (20–26 cores total). Significant *P* values in bold.

**Table 6:** Detection rates by lesion volume

	TB			eTB			sTB			iTB			TB + SB	
n/a = 28	*n*	% of TB+SB	95%CI	*n*	% of TB+SB	95%CI	*n*	% of TB+SB	95%CI	*n*	% of TB+SB	95%CI	*n*	% of TB+SB
≤**0.5 mL** ***N*** **= 237**														
Any prostate cancer	101	64	55.5–71.0	115	72	64.7–79.1	145	91	85.7–95.1	148	93	88.0–96.5	159	100
Gleason score ≥7	50	55	44.2–65.4	63	69	58.7–78.5	82	90	82.1–95.4	82	90	82.1–95.4	91	100
>**0.5 mL** ***N*** **= 222**														
Any prostate cancer	128	78	70.4–83.7	137	83	76.4–88.4	150	91	85.4–94.8	155	94	89.1–97.1	165	100
Gleason score ≥7	88	76	67.0–83.3	95	82	73.7–88.4	109	94	88.0–97.5	107	92	85.8–96.4	116	100
***P*** **value**														
Any prostate cancer	**0.007**			**0.023**			1.000			0.824				
Gleason score ≥7	**0.002**			**0.047**			0.432			0.626				

TB, two cores; eTB, four cores total; iTB, 14 cores total; sTB, 10–20 cores total depending on location; TB+SB, standard TB plus 18–24 SB sector cores (20–26 cores total). Significant *P* values in bold.

## Discussion

The two‐core TB performed poorly, detecting only 67% of Gleason score ≥7 cancers detected by the gold standard TB+SB approach. To achieve an overall detection rate of >90% for Gleason score ≥7 cancers, a saturation TB (sTB) was needed. This approach, with two TB plus two sector biopsies plus two cores from each of the adjacent Ginsburg sectors, would reduce total biopsy core numbers from 20–26 to 10–20, depending on the target lesion location. An iTB approach also detected >90% of Gleason score 7–10 cancers with two TB plus 12 ipsilateral systematic cores.

Our results for the four‐core eTB (76% sensitivity for Gleason score ≥7) closely match those reported by Bryk et al. 13, 27 using a four‐core transrectal TB approach (73% sensitivity for Gleason score ≥7) and Mischinger et al. 13, 27 for a four‐core robot‐assisted transperineal keyhole biopsy (80% sensitivity for Gleason score ≥7). Calio et al. 14 reported a higher sensitivity of 94% for Gleason score 7–10 with four‐core transrectal TB, but this may reflect differences between study populations; our present study showed that MRI lesion probability, lesion location and volume, and prostate volume all potentially affect the results of targeted biopsies.

Our sTB and iTB models both had >90% sensitivity for Gleason score 7–10 cancer, similar to the results Bryk et al. 13 reported with an ipsilateral 4+6 core biopsy model. This may be due to overcoming potential software fusion errors, being notably similar to the performance of direct in‐bore prostate biopsy with 2–4 cores 6. As both sTB and iTB performed very similarly in our present study, we cannot recommend using one over the other. The sTB approach has the advantage that it would allow lesser numbers in a considerable amount of patients, as lesions in the peripheral parts of the prostate are more common. Centrally positioned lesions in the middle of the transition zone and therefore requiring 20 cores are relatively rare. However, choosing the right number and location of cores based on the location of the lesion may require a higher level of skill and expertise and therefore more training. The iTB approach standardises the number of cores, but it still requires the skill to distribute the SB ones in an optimal pattern. One could argue that one would choose the iTB approach if there is a high turnaround of trainees who usually deliver the service of prostate biopsies. Experts may choose the sTB approach. Either approach would benefit from software guidance, which is already available on most leading fusion‐biopsy machines. However, not every hospital or even health economy can afford this equipment. Although it may be tempting, the present study cannot claim to give the answer but some good evidence to support a decision‐making process or in fact initiate trials which compare one or the other.

But are TB models with even fewer biopsy cores also safe? In our present study, a four‐core eTB found only 76% of Gleason score 7–10 cancers in the general study population but performed well when patients met certain criteria: 100% sensitivity for Gleason score ≥7 if Likert 5 cancer probability, lesion volume >0.5 mL, anterior lesion localisation, and prostate volume ≤45 mL. The better performance for anterior lesions may relate to the anterior portion of the prostate being less mobile and thereby easier to target than the posterior gland, whilst big lesions in small prostates are easier to target 28. Likert 5 lesions might need fewer cores because they are usually larger and therefore easier to hit, whereas smaller lesions might suffer more from fusion errors and might require ‘peppering’ of the target area with more cores. Conversely, smaller prostate volumes lead to a denser sampling of prostate tissue and the respective target area by rather few adjacent SB cores.

Only 5% of patients in our present study would have avoided a diagnosis of a Gleason score 6 cancer if only a sTB had been done, which is considerably less than the 19% reported by Bryk et al. 13 but comparable to the 9% reported in the PRECISION trial 4. Further reduction of systematic cores, for instance acquiring only one core per adjacent sector may be feasible. Complications of the Ginsburg technique have been reported previously 29; there were no serious complications or hospital readmissions. As the present analysis is a statistical modelling of data from patients who all had a full Ginsburg biopsy, we cannot provide any data on procedure time, discomfort or complications of the reduced biopsy models. Reducing the number of cores would likely reduce workload for pathologists, shorten procedure time, and minimise patient discomfort and complications 30. Our present results suggest that a reduction to four TBs plus 6–16 surrounding cores could safely replace the standard 20–26‐core Ginsburg template. Transperineal prostate biopsies are traditionally performed under general anaesthesia due to poor tolerance. A reduction to four TB cores under certain circumstances may make performing procedures under local anaesthesia achievable. Indeed, some case series have suggested that transperineal template prostate biopsy under local anaesthesia is feasible 31.

A limitation of our present study is its lack of prostatectomy specimens for definite histological verification. However, our present study design did make it possible to include all men in the analysis, including those with benign outcomes, not only those who had significant cancer, similar to the PROstate Magnetic resonance Imaging Study (PROMIS) study 32. Radtke et al. 17 have shown that the Ginsburg template reliably detected 97% of Gleason score ≥7 cancers later found at prostatectomy. Regarding overall quality of MRI reporting and combined biopsy, our Gleason score 7–10 detection rate in Likert 3 lesions of 20% compares to 21% in the PROMIS study 32, 17% in the MRI First study 5, and 18% in the 4M study 6, whilst being only slightly higher than the 12% in the PRECISION trial 4, which could be due to our mixed study population. The detection in our present study of 56% Gleason score 7–10 cancers in men with suspicious MRI (Likert 4–5) by the full Ginsburg template is slightly lower than previously reported for an initial mapping biopsy 32 and primary transperineal MR/US fusion TB 11, but comparable to those for a repeat biopsy population 10. This may by explained by the mixed study population assessed, with both biopsy‐naïve men and patients with previous negative biopsies or low‐grade disease assessed, as well as MRI evaluation performed during the transition period between PI‐RADS version 1 and 2. Only men with a single MRI lesion were included in our present study, so the results may not be generalised to men with multiple MRI lesions. Also, our present results were derived from data at a specialised tertiary care prostate centre with extensive fusion biopsy experience of both uroradiologists and urologists. Inter‐reader variability of MRI needs to be taken into account 33, 34 and optimal communication of lesion localisation needs to be ensured between radiologist and operator, especially if only a four‐core biopsy is undertaken, ideally with target outlining performed by the reporting radiologist. Due to the necessary learning curve 35, it is likely that less experienced operators will need to sample more SB cores, particularly from large prostates and small lesions. Once high negative and positive predictive values are ensured by continuous quality management, including good communication between radiologist and operator, reducing the core number seems reasonable. As part of quality assurance, any mismatch in imaging with high probability and benign histopathology should be reviewed in multidisciplinary meetings for further management decisions.

## Conclusions

sTB detected Gleason score 7–10 cancer in 25% more of the men than a two‐core TB approach, and in almost as many men (91%) as the 20–26‐core TB+SB, whilst needing only 10–20 cores. A four‐core eTB may suffice for large, highly suspicious anterior lesions in small or slightly enlarged prostates.

## Funding

Nienke L. Hansen has received a research grant from RWTH Aachen University Hospital (Aachen, Germany) and a Philips Healthcare Germany clinical research fellowship (Hamburg, Germany) and has received speaker fees from Guerbet. Tristan Barrett acknowledges support from Cancer Research UK, National Institute of Health Research (NIHR) Cambridge Biomedical Research Centre (BRC), Cancer Research UK and the Engineering and Physical Sciences Research Council Imaging Centre in Cambridge and Manchester and the Cambridge Experimental Cancer Medicine Centre. Anne Warren acknowledges support from the NIHR Cambridge BRC, UK. Christof Kastner acknowledges that he has received speaker or mentorship fees from Siemens Healthcare and MedCom GmbH. The Department of Urology, Addenbrooke's Hospital, Cambridge, UK, also received sponsorship of various industry for organising Prostate MRI workshops.

## Conflict of Interest

The authors declare that they have no conflict of interest.

## Disclaimers

None.

## Supporting information


**Table S1.** Gleason score of biopsy (*n* = 487).
**Table S2.** Detection rates by sector location (outer vs inner sectors).
**Table S3.** Detection rates in the subgroup of large lesions in the anterior sectors of a small prostate.
