# Supplementary material for: Optimising the number of cores for magnetic resonance imaging‐guided targeted and systematic transperineal prostate biopsy
Source: BJU Int. 2019 Aug 1;125(2):260–9. doi: 10.1111/bju.14865 (PMC8641376; doi:10.1111/bju.14865)
Supplement: Supplementary file 1 — Table S1. Gleason score of biopsy (n = 487). Table S2. Detection rates by sector location (outer vs inner sectors). Table S3. Detection rates in the subgroup of large lesions in the anterior sectors of a small prostate. [file BJU-125-260-s001.docx]

**Supplementary Material**

**Table S1: Gleason score of biopsy (n=487)**

TB = targeted biopsy (2 cores); eTB = “Extended target biopsy” (4 cores total); sTB = “Saturation target biopsy” (10-20 cores total depending on location); iTB = “Ipsilateral target biopsy” (14 cores total); TB+SB = standard targeted biopsy plus 18-24 systematic sector cores (20-26 cores total)

|  | TB | | eTB | | sTB | | iTB | | Total TB+SB | |
| --- | --- | --- | --- | --- | --- | --- | --- | --- | --- | --- |
| Gleason Score | n | % of 487 | n | % of 487 | n | % of 487 | n | % of 487 | n | % of 487 |
| benign | 241 | 49% | 218 | 45% | 173 | 36% | 164 | 34% | 142 | 29% |
| 3+3 | 97 | 20% | 100 | 21% | 112 | 23% | 122 | 25% | 124 | 25% |
| 3+4 | 100 | 21% | 113 | 23% | 125 | 26% | 127 | 26% | 133 | 27% |
| 4+3 | 21 | 4% | 23 | 5% | 37 | 8% | 36 | 7% | 44 | 9% |
| 8 | 13 | 3% | 17 | 3% | 18 | 4% | 17 | 3% | 21 | 4% |
| 9 | 15 | 3% | 16 | 3% | 21 | 4% | 20 | 4% | 22 | 5% |
| 10 | 0 | 0% | 0 | 0% | 1 | 0% | 1 | 0% | 1 | 0% |
| PCa | 246 | 51% | 269 | 55% | 314 | 65% | 323 | 66% | 345 | 71% |
| PCa ≥3+4 | 149 | 31% | 169 | 35% | 202 | 41% | 201 | 41% | 221 | 45% |
| PCa ≥4+3 | 49 | 10% | 56 | 11% | 77 | 16% | 74 | 15% | 88 | 18% |

**Table S2: Detection rates by sector location (Outer vs inner sectors)**

TB = targeted biopsy (2 cores); eTB = “Extended target biopsy” (4 cores total); sTB = “Saturation target biopsy” (10-20 cores total depending on location); iTB = “Ipsilateral target biopsy” (14 cores total); TB+SB = standard targeted biopsy plus 18-24 systematic sector cores (20-26 cores total)

CI = Confidence interval; PPV = positive predictive value

|  |  |  | **TB** | | | **eTB** | | | **sTB** | | | **iTB** | | | **TB+SB** | |
| --- | --- | --- | --- | --- | --- | --- | --- | --- | --- | --- | --- | --- | --- | --- | --- | --- |
|  |  |  | n | % of TB+SB | 95%CI | n | % of TB+SB | 95%CI | n | % of TB+SB | 95%CI | n | % of TB+SB | 95%CI | n | % of TB+SB |
| **Outer sectors 1L 1M 2L 3L 4L 4M 5L 6M 6L**  **N=442** | | **Any PCa** | 227 | 72% | [66.8-76.9] | 250 | 79% | [74.5-83.7] | 285 | 91% | [86.7-93.5] | 295 | 94% | [90.4-96.1] | 315 | 100% |
|  |  | **GS** ≥7 | 139 | 68% | [61.3-74.5] | 159 | 78% | [71.6-83.4] | 185 | 91% | [85.8-94.3] | 184 | 90% | [85.3-93.9] | 204 | 100% |
| **Inner sectors 2M 5M**  **N=45** | | **Any PCa** | 19 | 63% | [43.9-80.1] | 19 | 63% | [43.9-80.1] | 29 | **97%** | **[82.8-99.9]** | 28 | 93% | [77.9-99.2] | 30 | 100% |
|  |  | **GS** ≥7 | 10 | 59% | [32.9-81.6] | 10 | 59% | [32.9-81.6] | 17 | **100%** | **[83.8-1.00]** | 17 | **100%** | **[83.8-1.00]** | 17 | 100% |
| **p-value** | | **Any PCa** | 0.398 |  |  | 0.062 |  |  | 0.339 |  |  | 1.000 |  |  |  |  |
| **p-value** | | **GS ≥7** | 0.591 |  |  | 0.131 |  |  | 0.373 |  |  | 0.241 |  |  |  |  |

**Table S3: Detection rates in the subgroup of large lesions in the anterior sectors of a small prostate.**

TB = targeted biopsy (2 cores); eTB = “Extended target biopsy” (4 cores total); sTB = “Saturation target biopsy” (10-20 cores total depending on location); iTB = “Ipsilateral target biopsy” (14 cores total); TB+SB = standard targeted biopsy plus 18-24 systematic sector cores (20-26 cores total)

CI = Confidence interval; PPV = positive predictive value

|  |  | **TB**  **2 cores** | | | **eTB**  **4 cores** | | | **sTB**  **10-14 cores** | | | **iTB**  **14 cores** | | | **TB+SB**  **20-26 cores** | | |
| --- | --- | --- | --- | --- | --- | --- | --- | --- | --- | --- | --- | --- | --- | --- | --- | --- |
|  |  | n | % of TB+SB | 95%CI | n | % of TB+SB | 95%CI | N | % of TB+SB | 95%CI | N | % of TB+SB | 95%CI | n | % of TB+SB | *PPV* |
| **Likert 4**  **N=15** | **Any PCa** | 11 | **92%** | **[61.5-99.8]** | 12 | **100%** | **[77.9-1.00]** | 12 | **100%** | **[77.9-100]** | 12 | **100%** | **[77.9-100]** | 12 | 100% | *80%* |
|  | **GS** ≥7 | 5 | **83%** | **[35.9-99.6]** | 5 | **83%** | **[35.9-99.6]** | 6 | **100%** | **[60.7-100]** | 5 | **83%** | **[35.9-99.6]** | 6 | 100% | *40%* |
| **Likert 5**  **N=33** | **Any PCa** | 30 | **94%** | **[79.2-99.2]** | 31 | **97%** | **[83.8-99.9]** | 32 | **100%** | **[91.1-100]** | 31 | **97%** | **[83.8-99.9]** | 32 | 100% | *97%* |
|  | **GS** ≥7 | 25 | **96%** | **[80.4-99.9]** | 26 | **100%** | **[89.1-100]** | 26 | **100%** | **[89.1-100]** | 26 | **100%** | **[89.1-100]** | 26 | 100% | *79%* |
| **Likert 4-5**  **N=48** | **Any PCa** | 41 | 93% | [81.3-98.6] | 43 | **98%** | **[88.0-99.9]** | 44 | **100%** | **[93.4-100]** | 43 | **98%** | **[88.0;-99.9]** | 44 | 100% | *92%* |
|  | **GS** ≥7 | 30 | 94% | [79.2-99.2] | 31 | **97%** | **[83.8-99.9]** | 32 | **100%** | **[91.1-100]** | 31 | **97%** | **[83.8-99.9]** | 32 | 100% | *67%* |
